# The UPMC OPTIMISE-C19 (OPtimizing Treatment and Impact of Monoclonal antIbodieS through Evaluation for COVID-19) trial: a structured summary of a study protocol for an open-label, pragmatic, comparative effectiveness platform trial with response-adaptive randomization

**DOI:** 10.1186/s13063-021-05316-3

**Published:** 2021-05-25

**Authors:** David T. Huang, Erin K. McCreary, J. Ryan Bariola, Richard J. Wadas, Kevin E. Kip, Oscar C. Marroquin, Stephen Koscumb, Kevin Collins, Judith A. Shovel, Mark Schmidhofer, Mary Kay Wisniewski, Colleen Sullivan, Donald M. Yealy, Meredith Axe, David A. Nace, Ghady Haidar, Tina Khadem, Kelsey Linstrum, Graham M. Snyder, Christopher W. Seymour, Stephanie K. Montgomery, Bryan J. McVerry, Lindsay Berry, Scott Berry, Russell Meyers, Alexandra Weissman, Octavia M. Peck-Palmer, Alan Wells, Robert Bart, Debbie L. Albin, Tami Minnier, Derek C. Angus

**Affiliations:** 1 Department of Emergency Medicine, University of Pittsburgh School of Medicine, 606B Scaife Hall, Pittsburgh, PA 15213 USA; 2 Department of Critical Care Medicine, University of Pittsburgh School of Medicine, Pittsburgh, PA USA; 3 Division of Infectious Diseases, Department of Medicine, University of Pittsburgh School of Medicine, Pittsburgh, PA USA; 4 Clinical Analytics, UPMC, Pittsburgh, PA USA; 5 Wolff Center, UPMC, Pittsburgh, PA USA; 6 Division of Cardiology, Department of Medicine, University of Pittsburgh School of Medicine, Pittsburgh, PA USA; 7 UPMC Health System Office of Healthcare Innovation, Pittsburgh, PA USA; 8 Division of Geriatric Medicine, Department of Medicine, University of Pittsburgh, Pittsburgh, PA USA; 9 Division of Pulmonary, Allergy, and Critical Care Medicine, Department of Medicine, University of Pittsburgh School of Medicine, Pittsburgh, PA USA; 10 Berry Consultants, Austin, TX USA; 11 Department of Pathology, University of Pittsburgh School of Medicine, Pittsburgh, PA USA; 12 UPMC Health Services Division, Pittsburgh, PA USA; 13 UPMC Supply Chain Management/HC Pharmacy, Pittsburgh, PA USA

**Keywords:** COVID-19, pragmatic trial, randomised controlled trial, protocol, monoclonal antibodies, bamlanivimab, etesevimab, casirivimab, imdevimab

## Abstract

**Objectives:**

The primary objective is to evaluate the comparative effectiveness of COVID-19 specific monoclonal antibodies (mABs) with US Food and Drug Administration (FDA) Emergency Use Authorization (EUA), alongside UPMC Health System efforts to increase patient access to these mABs.

**Trial design:**

Open-label, pragmatic, comparative effectiveness platform trial with response-adaptive randomization

**Participants:**

We will evaluate patients who meet the eligibility criteria stipulated by the COVID-19 mAB EUAs who receive mABs within the UPMC Health System, including infusion centers and emergency departments. EUA eligibility criteria include patients with mild to moderate COVID-19, <10 days of symptoms, and who are at high risk for progressing to severe COVID-19 and/or hospitalization (elderly, obese, and/or with specific comorbidities). The EUA criteria exclude patients who require oxygen for the treatment of COVID-19 and patients already hospitalized for the treatment of COVID-19. We will use data collected for routine clinical care, including data entered into the electronic medical record and from follow-up calls.

**Intervention and comparator:**

The interventions are the COVID-19 specific mABs authorized by the EUAs. All aspects of mAB treatment, including eligibility criteria, dosing, and post-infusion monitoring, are as per the EUAs. As a comparative effectiveness trial, all patients receive mAB treatment, and the interventions are compared against each other.

When U.S. government mAB policies change (e.g., FDA grants or revokes EUAs), UPMC Health System policies and the evaluated mAB interventions will accordingly change. From November 2020 to February 2021, FDA issued EUAs for three mAB treatments (bamlanivimab; bamlanivimab and etesevimab; and casirivimab and imdevimab), and at trial launch on March 10, 2021 we evaluated all three. Due to a sustained increase in SARS-CoV-2 variants in the United States resistant to bamlanivimab administered alone, on March 24, 2021 the U.S. Government halted distribution of bamlanivimab alone, and UPMC accordingly halted bamlanivimab monotherapy on March 31, 2021. On April 16, 2021, FDA revoked the EUA for bamlanivimab monotherapy. At the time of manuscript submission, we are therefore evaluating the two mAB treatments authorized by EUAs (bamlanivimab and etesevimab; and casirivimab and imdevimab).

**Main outcomes:**

The primary outcome is total hospital free days (HFD) at 28 days after mAB administration,

calculated as 28 minus the number of days during the index stay (if applicable – e.g., for patients admitted to hospital after mAB administration in the emergency department) minus the number of days readmitted during the 28 days after treatment. This composite endpoint captures the number of days from the day of mAB administration to the 28 days thereafter, during which the patient is alive and free of hospitalization. Death within 28 days is recorded as -1 HFD, as the worst outcome.

**Randomisation:**

We will start with equal allocation. Due to uncertainty in sample size, we will use a Bayesian adaptive design and response adaptive randomization to ensure ability to provide statistical inference despite variable sample size.

When mABs are ordered by UPMC physicians as a generic referral order, the order is filled by UPMC pharmacy via therapeutic interchange. OPTIMISE-C19 provides the therapeutic interchange via random allocation. Infusion center operations teams and pharmacists use a mAB assignment application embedded in the electronic medical record to determine the random allocation.

**Blinding (masking):**

This trial is open-label. However, outcome assessors conducting follow-up calls at day 28 are blinded to mAB assignment, and investigators are blinded to by-mAB aggregate outcome data until a statistical platform trial conclusion is reached.

**Numbers to be randomised (sample size):**

Sample size will be determined by case volume throughout the course of the pandemic, supply of FDA authorized mABs, and by that needed to reach a platform trial conclusion of inferiority, superiority, or futility of a given mAB. The trial will continue as long as more than one mAB type is available under EUA, and their comparative effectiveness is uncertain.

**Trial Status:**

Protocol Version 1.0, February 24, 2021.

Recruitment began March 10, 2021 and is ongoing at the time of manuscript submission. The estimated recruitment end date is February 22, 2022, though the final end date is dependent on how the pandemic evolves, mAB availability, and when final platform trial conclusions are reached. As noted above, due to U.S. Government decisions, UPMC Health System halted bamlanivimab monotherapy on March 31, 2021.

**Trial registration:**

ClinicalTrials.gov Identifier: NCT04790786. Registered March 10, 2021

**Full protocol:**

The full protocol is attached as an additional file, accessible from the Trials website (Additional file 1). In the interest in expediting dissemination of this material, the familiar formatting has been eliminated; this Letter serves as a summary of the key elements of the full protocol.

**Supplementary Information:**

The online version contains supplementary material available at 10.1186/s13063-021-05316-3.

## Supplementary Information

**Additional file 1.**

## Data Availability

Data will be made available from the author on reasonable request and pursuant to contractual agreements, via contacting David T. Huang at huangdt@upmc.edu.

