# Supplementary material for: The UPMC OPTIMISE-C19 (OPtimizing Treatment and Impact of Monoclonal antIbodieS through Evaluation for COVID-19) trial: a structured summary of a study protocol for an open-label, pragmatic, comparative effectiveness platform trial with response-adaptive randomization
Source: Trials. 2021 May 25;22:363. doi: 10.1186/s13063-021-05316-3 (PMC8144687; doi:10.1186/s13063-021-05316-3)
Supplement: Supplementary file 1 — Additional file 1. [file 13063_2021_5316_MOESM1_ESM.pdf]

UPMC Antibody Treatment and Evaluation Center

A pragmatic evaluation of monoclonal antibody treatments in participants with COVID-19 illness

Version 1.0

February 24, 2021

**Summary**	
Background	• FDA Emergency Use Authorization (EUA) exists for multiple monoclonal antibodies (mAB) to treat COVID-19; the EUAs stipulate eligibility criteria, patient-physician communication, and clinical monitoring.<br>• UPMC provides mABs as routine care; physicians order a mAB infusion and pharmacies assign whichever mAB is available under a therapeutic interchange approach. If scarcity exists, a lottery system is used.<br>• Physicians review with patients the EUA Fact Sheet for each mAB, and explain they could be assigned any of the EUA-governed mABs.
Approach	• Structure the therapeutic interchange policy and lottery system using a UPMC pharmacy embedded assignment system that allows a comparative effectiveness evaluation of the multiple mABs.<br>• Collect data from clinically performed UPMC processes and EUA requirements for routine care.
Treatments	• Monoclonal antibodies (mAB) for COVID-19
Inclusion Criteria	These criteria are as per the FDA EUAs for COVID-19 mABs as of February 24, 2021.<br><br>• Adult and pediatric patients (12 years of age and older weighing at least 40 kg) with a positive SARS-CoV-2 antigen or PCR test and within 10 days of symptom onset, and high risk of disease progression<br>• High risk is defined as patients who meet at least one of the following criteria:<br>○ Body Mass Index (BMI) ≥35<br>○ Have Chronic Kidney Disease<br>○ Have diabetes<br>○ Have immunosuppressive disease<br>○ Are currently receiving immunosuppressive treatment<br>○ Are ≥65 years of age<br>○ Are ≥55 years of age AND have:<br>○ cardiovascular disease, OR<br>○ hypertension, OR<br>○ chronic obstructive pulmonary disease/other chronic respiratory disease<br>○ Are 12-17 years of age AND have:<br>○ BMI ≥ 85th percentile for their age and gender based on CDC growth charts, OR<br>○ sickle cell disease, OR<br>○ congenital or acquired heart disease, OR<br>○ neurodevelopmental disorders (eg, cerebral palsy), OR<br>○ a medical-related technological dependence, for example, tracheostomy or gastrostomy), OR<br>○ asthma, reactive airway or other respiratory disease that requires daily medication for control.
Exclusion Criteria	These criteria are as per the FDA EUAs for COVID-19 mABs as of February 24, 2021.<br><br>• Are hospitalized for the treatment of COVID-19<br>• Require oxygen therapy for the treatment of COVID-19

	• Require an increase in baseline oxygen flow rate due to COVID-19 in those on chronic oxygen therapy due to underlying non-COVID-19 related comorbidity<br>• Have a known hypersensitivity to any antibody ingredient
Primary evaluation metric	Total hospital free days at 28 days

Table of Contents

1. ABBREVIATIONS 6
2. BACKGROUND and RATIONALE 7
2.1. BACKGROUND 7
2.2. RATIONALE 8
3. OBJECTIVES AND METRICS 9
3.1. OBJECTIVES 9
3.2. METRICS 9
4. DESIGN 10
4.1. POPULATION 10
4.1.1. INCLUSION CRITERIA 10
4.1.2. EXCLUSION CRITERIA 11
5. EVALUATED TREATMENTS 12
5.1. BAMLANIVIMAB 12
5.2. BAMLANIVIMAB and ETESVIMAB 12
5.3. CASIRIVIMAB and IMDEVIMAB 12
5.4. CONCOMITANT THERAPY 13
6. CONDUCT 14
6.1. DATA COLLECTION 14
6.2. BIOSPECIMENS 15
6.3. ANTIBODY ADMINISTRATION 15
6.4. mAB assignment 15
7. STATISTICAL CONSIDERATIONS 16
7.1. STRATA 16
7.2. NUMBER of PARTICIPANTS 16
7.3. STATISTICAL ANALYSIS 16
8. ETHICAL CONSIDERATIONS 17
8.1. DATA MONITORING 17
8.2. CONSENT 17
8.3. ADVERSE EVENTS and SERIOUS ADVERSE EVENTS 17
8.3.1. SAFETY and RISK MITIGATION 17
8.3.2. MANAGEMENT of INFUSION REACTIONS 18
9. EXHIBITS 18

1. ABBREVIATIONS

AE	Adverse Events
B	bamlanivimab
B + E	bamlanivimab and etesevimab
BMI	Body Mass Index
C + I	casirivimab + imdevimab
CDC	Centers for Disease Control and Prevention
COPD	Chronic Obstructive Pulmonary Disease
COVID-19	Coronavirus disease 2019
CVD	Cardiovascular Disease
EUA	Emergency Use Authorization
FDA	Food and Drug Administration
HHS	Health and Human Services
HFD	Hospital Free Days
HTN	Hypertension
kDa	Kilodaltons
IgG1	Immunoglobulin G1
KG	Kilograms
mAB	Monoclonal Antibodies
PCR	Polymerase Chain Reaction
SAEs	Serious Adverse Events
SARS-CoV-2	Severe Acute Respiratory Syndrome Coronavirus 2
UATRC	UPMC Antibody Treatment and Evaluation Center

2. BACKGROUND and RATIONALE

2.1. BACKGROUND

While COVID-19 vaccination will reduce COVID-19-related morbidity and mortality, the learned immune response may vary between individuals. This means interventions such as monoclonal antibodies (mAB) will still be needed to prevent progression of COVID-19 illness. Monoclonal antibodies seek to mimic or enhance the natural immune system response against a pathogen and are often used in the care of patients with cancer or infection.

For viral infections, mABs are created by exposing a white blood cell to a particular viral protein, which is then cloned to mass produce antibodies to target that virus. For SARS-CoV-2, the virus that causes COVID-19, IgG1 mABs target the spike protein of SARS-CoV-2 and block viral attachment and entry into cells.

The SARS-CoV-2 mABs bamlanivimab and etesevimab, and the REGN-COV2 combination (casirivimab + imdevimab) reduce nasopharyngeal viral burden plus clinical outcomes including future emergency department visits and hospitalizations (Weinreich 33332778 NEJM, Gottlieb 33475701). Each received FDA Emergency Use Authorization (EUA) for use in selected populations (**Exhibit**). Additional trials of pre-exposure prophylaxis (NCT04497987) and other applications are underway, and additional mABs are in development.

The trials demonstrated the greatest impact of the REGN-COV2 dual therapy among patients who lacked neutralizing antibodies against SARS-CoV-2 at baseline and in those with high nasopharyngeal viral loads. Additionally, few patients in the bamlanivimab/etesevimab trial developed treatment-emergent SARS-CoV-2 resistance. This latter phenomenon may further enhance the need for therapies given the recent emergence of SARS-CoV-2 variants that may escape vaccination. However, the relative effectiveness of each mAB compared to the other is unknown, as is their effectiveness for emerging virus variants.

This Appendix to the UPMC Pilot Core (PittPro 20040210) describes the approach of the UPMC Antibody Treatment and Evaluation Center. We will conduct a pragmatic evaluation of monoclonal antibody treatments in participants with COVID-19 illness, starting with the patient population approved under the current FDA mAB EAUs.

2.2. RATIONALE

As of February 2021, there are over 60,000 new cases of COVID-19 diagnosed daily in the US https://covid.cdc.gov/covid-data-tracker/#trends_dailytrendscases, with over 7000 daily COVID-19 related hospital admissions Microsoft Power BI (powerbigov.us). Although case volumes are currently declining, COVID-19 remains a significant public health threat.

Despite the EUAs, the clinical use of mABs is low due in part to lack of patient access, complexities in drug allocation, and lack of knowledge among providers are contributing factors. Further, the comparative effectiveness of different mABs is unknown and not yet directly studied. The National Academies of Sciences, Engineering, and Medicine recently called for expanded access and clinical use of mABs, noting it is "critical to collect data and evaluate whether they are working as predicted".

This evaluation seeks to expand access to mABs at UPMC and determine their relative effects versus each other, starting with those governed by EUAs.

3. OBJECTIVES AND METRICS

3.1. OBJECTIVES

The primary objective is to evaluate the clinical and biological effect of multiple monoclonal antibodies (mABs) in patients with COVID-19.

The primary hypothesis is clinical and biological effect will vary between mABs, by SARS-CoV-2 variants, and patient characteristics.

3.2. METRICS

The primary evaluation metric is total hospital free days (HFD) at 28 days after mAB receipt calculated as 28 minus the number of days during the index stay minus the number of days readmitted during the 28 days after treatment. Death within 28 days is recorded as -1 HFD.

Secondary evaluation metrics include:

- All-cause and all-location mortality at 28 and 90 days
- Emergency department visits at 28 days
- Organ-support free days at day 28
- Where feasible:
  - SARS-CoV-2 nasopharyngeal and plasma viral loads among participants from baseline and longitudinally through day 28
  - SARS-CoV-2 antibody titers, antibody neutralization, and other immune responses at baseline and longitudinally through day 28
  - Detection of SARS-CoV-2 variants through next-generation sequencing at baseline and longitudinally through day 28
  - Determining the duration of SAR-CoV-2 infectivity and non-culture surrogates for SARS-CoV-2 infectivity among patients with persistent nasopharyngeal swab viral shedding

4. DESIGN

We will conduct a pragmatic evaluation of participants with COVID-19 illness under existing UPMC processes for the clinical care of COVID-19 positive patients, including EUA requirements for mAB administration. A patient who presents to a UPMC facility and tests positive for COVID-19 will, as per current common care, be offered monoclonal antibodies. Data that are already collected according to UPMC procedures and EUA requirements are used for analysis.

4.1. POPULATION

We will evaluate patients that present to UPMC Emergency Departments, urgent care sites, infusions centers and other facilities that can or do provide mABs for COVID-19. As of February 24, 2021, there are 3 EUAs, with common inclusion and exclusion criteria, and we will evaluate patients that meet these criteria. As other antibodies become available, we will modify this evaluation submission.

4.1.1. INCLUSION CRITERIA

As per the current EUA criteria, the following patients are included:

- Adult (≥ 18 years old)
- Children ≥ 12 years old weighing at least 40 kg
- With a positive SARS-CoV-2 antigen or PCR test and within 10 days of symptom onset
- High risk of disease progression

High risk is defined as patients who meet at least one of the following criteria:

- A Body Mass Index (BMI) ≥ 35
- Have chronic kidney disease
- Have diabetes
- Have immunosuppressive disease
- Are currently receiving immunosuppressive treatment
- Are ≥ 65 years old
- Are ≥ 55 years of age AND have:
    - cardiovascular disease, OR

- hypertension, OR
- chronic obstructive pulmonary disease/other chronic respiratory disease

- Are 12-17 years of age AND have:
  - BMI ≥ 85th percentile for their age and gender based on CDC growth charts, OR
  - sickle cell disease, OR
  - congenital or acquired heart disease, OR
  - neurodevelopmental disorders (eg, cerebral palsy), OR
  - a medical-related technological dependence, for example, tracheostomy or gastrostomy), OR
  - asthma, reactive airway or other respiratory disease that requires daily medication for control

4.1.2. EXCLUSION CRITERIA

As per the current EUA criteria, the following are excluded:

- Are hospitalized for the treatment of COVID-19
- Require oxygen therapy for the treatment of COVID-19
- Require an increase in baseline oxygen flow rate due to COVID-19 in those on chronic oxygen therapy due to underlying non-COVID-19 related comorbidity
- Have a known hypersensitivity to any antibody ingredient

5. EVALUATED TREATMENTS

Patients will receive COVID-19 mABs governed by FDA EUAs, when their treating physician orders a mAB and they meet EUA criteria. Currently and under our examination, the treating physician do not choose a specific mAB product.

As of February 24, 2021, there are three such mABs as listed below.

5.1. BAMLANIVIMAB

Bamlanivimab is a human immunoglobulin G-1 (IgG1 variant) monoclonal antibody consisting of 2 identical light chain polypeptides composed of 214 amino acids each and 2 identical heavy chain polypeptides composed of 455 amino acids produced by a Chinese Hamster Ovary (CHO) cell line and molecular weight of 146 kDa.

5.2. BAMLANIVIMAB and ETESVIMAB

Bamlanivimab is a human immunoglobulin G-1 (IgG1 variant) monoclonal antibody consisting of 2 identical light chain polypeptides composed of 214 amino acids each and 2 identical heavy chain polypeptides composed of 455 amino acids produced by a Chinese Hamster Ovary (CHO) cell line and molecular weight of 146 kDa.

Etesevimab is a human IgG1 variant monoclonal antibody (mAb) consisting of 2 identical light chain polypeptides composed of 216 amino acids each and 2 identical heavy chain polypeptides composed of 449 amino acids produced by a Chinese Hamster Ovary (CHO) cell line and molecular weight of 145 kDa.

5.3. CASIRIVIMAB and IMDEVIMAB

Casirivimab, a human immunoglobulin G-1 (IgG1) monoclonal antibody (mAb), is covalent eterotetramer consisting of 2 heavy chains and 2 light chains produced by recombinant DNA technology in Chinese hamster ovary (CHO) cell suspension culture and has an approximate molecular weight of 145.23 kDa.

Imdevimab, a human IgG1 mAb, is a covalent heterotetramer consisting of 2 heavy chains and 2 light chains produced by recombinant DNA technology in Chinese hamster ovary (CHO) cell suspension culture and has an approximate molecular weight of 144.14 kDa.

5.4. CONCOMITANT THERAPY

All care and concomitant therapy are as per the treating providers.

6. CONDUCT

6.1. DATA COLLECTION

The EUAs require that healthcare facilities and providers report therapeutic information and utilization data through HHS Protect, Teletracking, or National Healthcare Safety Network as directed by the US Department of Health and Human Services.

We will collect data including baseline demographics and underlying conditions, results of SARS-COV-2 PCR or antibody testing, and initial care including mAB infusion completion. We will collect post-randomization healthcare encounters, including hospitalization, emergency department visits, ICU care, and other measures of healthcare utilization. We will use an electronic health record data collection process to augment existing UPMC data collection processes as necessary.

All data will be handled and secured as per University of Pittsburgh and UPMC data guidelines.

There will be no research activities involving direct interaction with subjects performed as part of this evaluation.

In addition to the primary and secondary outcome data referenced in this submission, data collected will include the below areas. All data will be abstracted directly from the electronic health record and handled anonymously.

- Which mAB was administered, including date, time, and infusion completion as well as the location of the infusion
- Demographics (including age, sex, race, body weight, vaccination status)
- Healthcare encounters, including hospital and ICU admission status if applicable
- Medication usage and doses
- Hospital and ICU admission status, if applicable
- Administration of medications related to COVID-19, if applicable
- Remnant blood availability
- Laboratory and microbiology data, including COVID-19 testing done for clinical purposes

6.2. BIOSPECIMENS

Where feasible, we will collect discarded remnant blood samples and nasal/oropharyngeal swab samples to quantify the viral load and host response to the virus. As noted under data collection, we will record laboratory and microbiology data performed for clinical purposes.

6.3. ANTIBODY ADMINISTRATION

Antibodies will be administered as per the EUAs and UPMC Pharmacy and Therapeutics policies. Providers will explain mAB risks and benefits and provide the EUA Fact Sheets for Patients, Parents and Caregivers as per EUA requirements.

6.4. mAB assignment

The COVID-19 mABs are currently routinely used at UPMC. Once any order for mAB infusion is approved by the UPMC system oversight group, the pharmacy provides whichever EUA-governed mAB is available under a therapeutic interchange approach. Ordering physicians review with the patient the EUA Fact Sheet for each mAB, and explain that the patient could receive any of the mABs governed by FDA EUAs.

If demand for mAB exceeds supply, UPMC has a lottery system to allot who receives the therapy once requested by a physician.

Our current proposal is a UPMC system quality improvement initiative, embracing and extending the current lottery system and therapeutic interchange policy for EUA-governed mABs for COVID-19 as follows:

1. The Physician orders mAB.

    a. If scarcity present and lottery system allow provision, proceed.

2. The Pharmacy fills order with one of the EUA-governed COVID-19 mABs using an embedded assignment system akin to current mAb provision. This system will allow a comparative effectiveness evaluation of the multiple mABs by effectively ensuring random allocation.

3. The Physician can agree to the assigned mAB or can request a specific mAB.

It is the treating physician's choice to accept the assigned mAB or not, and therefore patient consent for the mAB assignment is not required. Patients will be told which mAB they are receiving, along with an EUA Fact Sheet, as per EUA requirements.

7. STATISTICAL CONSIDERATIONS

7.1. STRATA

Predefined strata will include patients discharged home after infusion, patients admitted to hospital after infusion, prior vaccination, and if known, presence of virus variants of concern at baseline and presence of neutralizing antibodies to SARS-CoV-2 at baseline.

7.2. NUMBER of PARTICIPANTS

Sample size is determined by case volume throughout the course of the pandemic.

7.3. STATISTICAL ANALYSIS

The primary evaluation metric is the number of days free from hospitalization to day 28. We will finalize a statistical analysis plan which will consider mAB assigment, heterogeneity of treatment effect by patient characteristics and virus variants, and interaction with other treatments. Due to uncertainty in sample size, we will use a Bayesian adaptive design to ensure ability to provide statistical inference despite variable sample size.

8. ETHICAL CONSIDERATIONS

8.1. DATA MONITORING

Evaluation center leadership will regularly monitor monthly reports on enrollment, patient characteristics, and outcomes.

8.2. CONSENT

As per EUA requirements, physicians will discuss the risks and benefits of mABs and patients will consent to receive a mAB as part of usual care, should they desire mAB treatment. As per UPMC policy, the ordering physician reviews with patients the EUA Fact Sheet for each mAB and explain that the patient could receive any of the mABs governed by FDA EUAs.

8.3. ADVERSE EVENTS and SERIOUS ADVERSE EVENTS

The EUAs require providers and/or their designees report all medication errors and serious adverse events potentially related to the antibodies within seven calendar days from the onset of the event. Serious adverse events are defined as death, life-threatening event, inpatient hospitalization or prolongation of existing hospitalization, substantial disruption of ability to conduct normal life functions, a congenital anomaly/birth defect, or an intervention to prevent death, a life-threatening event, hospitalization, disability, or congenital anomaly.

The EUAs require adverse event reports be submitted to FDA MedWatch via one of multiple methods. Copies of all FDA MedWatch forms are also to be sent to the antibody manufacturer.

Thus, there already exist reporting requirements for UPMC associated with mAB prescription. We will track and record these reported data and adverse events by mAB assignment.

8.3.1. SAFETY and RISK MITIGATION

The EUAs stipulate warnings including hypersensitivity, clinical worsening, and side effects. As per EUA requirements, warnings will be communicated by providers to patients, adverse events will be reported as above, and post-infusion clinical monitoring will be done. Administration of mABs for patients with COVID-19 is routine care at UPMC, and their administration is not a research procedure.

8.3.2. MANAGEMENT of INFUSION REACTIONS

As per the EUAs, all participants should be monitored closely, as there is a risk of infusion reaction and hypersensitivity (including anaphylaxis) with any biological agent. Symptoms and signs that may occur as part of an infusion reaction include, but are not limited to fever, chills, nausea, headache, bronchospasm, hypotension, angioedema, throat irritation, rash including urticaria, pruritus, myalgia, and dizziness.

9. EXHIBITS

EUA fact sheets for health care providers

https://www.fda.gov/media/143603/download

https://www.fda.gov/media/143892/download

https://www.fda.gov/media/145802/download
